# Animal health as a function of farmer personality and attitude: using the HEXACO model of personality structure to predict farm-level seropositivity for *Fasciola hepatica* and *Ostertagia ostertagi* in dairy cows

**DOI:** 10.3389/fvets.2024.1434612

**Published:** 2024-10-02

**Authors:** Markus Leinmueller, Friedemann Adler, Amely Campe, Gabriela Knubben-Schweizer, Martina Hoedemaker, Christina Strube, Andrea Springer, Andreas W. Oehm

**Affiliations:** ^1^Clinic for Ruminants with Ambulatory and Herd Health Services, Ludwig-Maximilians-Universität Munich, Oberschleißheim, Germany; ^2^Department of Biometry, Epidemiology and Information Processing, WHO Collaborating Center for Research and Training for Health at the Human-Animal-Environment Interface, University of Veterinary Medicine Hannover, Hannover, Germany; ^3^Clinic for Cattle, University of Veterinary Medicine Hannover, Hannover, Germany; ^4^Institute for Parasitology, Centre for Infection Medicine, University of Veterinary Medicine Hannover, Hannover, Germany; ^5^Institute of Parasitology, University of Zurich, Zurich, Switzerland

**Keywords:** personality, attitude, cattle, parasite, HEXACO

## Abstract

**Introduction:**

Infections with *Fasciola hepatica* and *Ostertagia ostertagi* impinge upon dairy cow health and welfare and represent a major economic factor in livestock industry. Control measures largely rely on the use of anthelminthic drugs. However, reports of anthelmintic resistance necessitate sustainable approaches. Farmer characteristics such as attitude and personality are crucial for the implementation of control strategies and on-farm practices.

**Methods:**

In the present study, the HEXACO (Honesty-Humility, Emotionality, eXtraversion, Agreeableness, Conscientiousness, Openness to experience) model of personality structure, which conceptualizes human personality, was used to evaluate the relationship of farmer aspects with on-farm bulk tank milk seropositivity for *F. hepatica* and *O. ostertagi*. Moreover, information on farm structure, housing, management, and farmers’ attitude was collected in a face-to-face interview. Farm-level seropositivity for *F. hepatica* and *O. ostertagi* was predicted via elastic net regression.

**Results:**

Out of 193 farms housing 8,774 cows in the German Federal State of Bavaria, 47 farms (24.4%) were seropositive for *F. hepatica*, 77 farms (39.9%) for *O. ostertagi*, and 42 farms (21.8%) for both endoparasites. The model for *F. hepatica* seropositivity selected the covariates pasture access, *O. ostertagi* seropositivity, higher farmer conscientiousness, and organic farming as relevant predictors. Seropositivity for *O. ostertagi* was predicted by *F. hepatica* seropositivity, pasture access, organic farming, and farmers being neutral regarding their satisfaction with animal health on their farm. Higher values for the HEXACO factors extraversion and emotionality were inversely associated with *O. ostertagi* seropositivity.

**Discussion:**

The present work emphasizes the importance of farmer traits in regard to animal health and parasite occurrence. For the effective acceptance and implementation of sustainable control strategies for livestock helminth infections, it is crucial to consider these aspects to holistically address the challenges of managing parasitic diseases. Moreover, tailored communication strategies can be developed incorporating the understanding of individual stockman characteristics and subsequently ensuring encouragement of stakeholders.

## 1 Introduction

Parasitic infections represent a major problem in global livestock production (1, 2). Among parasitic helminths, *Fasciola hepatica* and *Ostertagia ostertagi* are the most important and widespread species in dairy cattle (3, 4) and have been associated with a decrease in productivity, economic viability, and wellbeing of animals (5–7). As for infections with *F. hepatica*, Schweizer et al. (6) have estimated an average reduction of 9% in weight gain for growing cattle, a 10% decrease in milk yield, an extension of the service period by 13 days, and an increase of 0.75 services per conception. A recent study calculated the reduction in milk yield, milk fat, and milk protein on seropositive dairy farms to be 1,206 kg, 22.9 kg, and 41.6 kg, respectively (5). Economic losses due to *O. ostertagi* have primarily been classified as indirect due to chronic parasitic gastroenteritis (8). A Mexican study determined an average reduction in milk production in the studied herds as high as 0.542 kg per cow per day (9). Even though infections often remain subclinical, Takeuchi-Storm et al. (10) emphasized that reductions in milk yield due to fasciolosis can be higher than those reported for clinical mastitis (11).

Helminth infections are challenging to control and the predominant strategy is to largely rely on the administration of anthelmintic drugs (12–14). In this very context it is yet crucial to be aware of the worldwide emergence of anthelmintic resistance to virtually all available compounds in both gastrointestinal nematodes and *F. hepatica* (15–17). This situation necessitates novel and integrated strategies to ensure sustainable parasite control and to guarantee animal health without relying on anthelmintic substances (18). Since the control of parasitic diseases as well as the implementation of intervention strategies on farm are the individual responsibilities of the farmer, the successful execution of management strategies to combat helminth infections is dependent on farmers’ decision-making (18, 19). Recent work has drawn attention to the importance of individual farmer characteristics and farm management practices in the implementation of on-farm intervention strategies and the inclination to incorporate new, targeted approaches to limit the impact of diseases on livestock (20–22). According to van den Borne et al. (23) farm performance frequently shows a stronger correlation with the personal traits of farmers, such as their personality or attitude, than with quantifiable management regimes. Hence, it is indispensable to understand which individual internal factors could influence how farmers decide or behave (22, 24).

Qualitative research with a focus on farmer traits has recently drawn attention as an integrated strategy to control helminth infections in livestock, and research to some extent has shifted its focus away from solely evaluating technical risk factors such as farming practices, housing conditions, or feeding regimes (24–26). Instead, growing emphasis has been placed on the farmer’s role and how their decisions could translate into animal health and productivity (22). Blending socio-psychological theories and methodologies with traditional epidemiological approaches has demonstrated its utility in examining the intentions and behaviors of cattle farmers (18, 26, 27). Within this particular framework, personality pertains to unique variations in how individuals typically think, feel, and act (28). These personality characteristics tend to stay fairly consistent once an individual surpasses the age of 30 (29). In contrast, attitude varies depending on the context and reflects a tendency or predisposition of people to respond toward an idea or situation or to interact with their surroundings (30). The HEXACO (honesty-humility, emotionality, extraversion, agreeableness, consciousness, openness to experience) model of personality structure stands as a widely recognized and validated tool for assessing personality in six distinct dimensions (31, 32). Evidence has suggested that personality and attitude can potentially impact various facets of dairy cattle welfare, performance, health, and farm management (22). This is of particular interest in helminth infections given their mostly subclinical nature and the fact that prevalence remains high in spite of decades of research on risk factors and intervention strategies (20, 33, 34). Policymakers and veterinarians have increasingly recognized the necessity of enhancing communication within the agricultural community regarding the handling of animal health risks (35, 36).

In order to provide tailored veterinary consultancy (24), veterinarians need to be aware of the individuals they are interacting with when offering expertise and consultation. This allows for the development and implementation of customized on-farm intervention programs that provide a sustainable framework for integrated parasite control and for the mitigation of adverse effects of parasitic infections in livestock. However, minimal evidence has been presented on the relevance of farmer personality in regard to helminth infections on dairy farms.

Therefore, the goal of the current study was to fill this gap and to understand if and to what extent farmer traits related to personality and attitude could predict the exposure to *F. hepatica* and *O. ostertagi* on dairy farms.

## 2 Materials and methods

### 2.1 Study area and sampling

From December 2016 to August 2019, a cross-sectional study was conducted on dairy farms in Germany (37). The German Ministry of Food and Agriculture (BMEL) initiated and funded the research project through the Federal Office for Agriculture and Food (BLE). In the present analysis, data collected from farms located in the south German federal state of Bavaria were evaluated. A total number of 260 dairy farms managing 11,539 cows were visited. Sample calculation for farm visits and farm selection process have been presented elsewhere (5, 38, 39). In brief, sample size was calculated given a power of 80% and a significance level of 5%, a disease prevalence of 50% was assumed, which resulted in 250 farms to be visited (40). Farm selection was random taking administrative district as well as herd size (number of dairy cows) into account, i.e., farms were randomly selected according to herd size within their respective administrative district and the study team was unaware of the identity of sampled farms during the sampling process. The national animal information database (Herkunftssicherungs-und Informationssystem für Tiere, HIT) and farm data from the Association for Milk Testing (Milchpruefring Bayern e. V.) provided information for sampling. From these sources, farms were randomly sampled. At first 1,250 selected farms were invited to participate by a letter explaining the purpose of the study. Farm managers had to voluntarily get in touch with the study team to arrange a date of the farm visit. Because initial response rate was below 10%, another 3,160 farms were sampled and invited (41). Upon participation, farmers were asked to give their written consent for data inspection and evaluation. All information related to farm characteristics and internal information was handled anonymously and with the utmost care in alignment with the German and European data protection legislation.

### 2.2 On-farm data collection

Data were collected by trained veterinarians on the animal and on the farm-level using questionnaires and data entry forms to cover an extensive selection of farm structure, housing, and management related aspects. Questionnaires and data entry forms had been developed prior to the start of the study and were evaluated during a three-day seminar of the whole study team. Subsequently, these forms underwent continued evaluation and discussion during a three-month pilot phase prior to commencement of data collection. During this period, three volunteering pilot farms brought forward by study veterinarians were visited to test and refine the data collection procedures. In addition to data collection and interviews, feasibility and appropriateness of the developed questionnaires and data entry forms were recorded and subjected to group discussions. Refinements of the documents and data collection procedures could hence be ensured. Subsequent feedback from farmers enabled appropriate modification. Standard operating procedures were developed prior to the assessment in order to minimize observer bias (42).

Face-to-face interviews were conducted between one of the study veterinarians at a time and the person responsible or jointly responsible for making management decisions on farm (43). Special attention was paid to asking questions in an open manner by veterinarians simply reading the questions out loud in order to prevent socially desirable answers rather than the true opinion. Furthermore, farmers were clearly informed that answers would be documented descriptively, analyzed across herds with complete anonymity and that tracing back answers to an individual farmer would not be possible. The interview consisted of four parts: Farmers were initially asked several questions about their own and their farm’s background (e.g., farming type) in order to build rapport. The second part consisted of questions to record farmers’ attitude toward their work and the animals (e.g., “it is important to me to show patience with my animals”). Farmers were asked to rate statements on a 5-point Likert scale (44): strongly disagree, disagree, neutral, agree, strongly agree. Each statement also contained “not stated” in case a farmer chose not to provide an answer. The responses were categorized into three distinct groups to reflect overall attitude toward each statement: Specifically, responses of “strongly disagree” and “disagree” were combined into a single category labeled “disagree/negative response.” Responses of “agree” and “strongly agree” were similarly combined into a single category labeled “agree/positive response.” This categorization was designed to simplify the analysis by grouping similar responses together, to incorporate a sufficient number of observations in each group and providing a clearer picture of the overall sentiment toward each statement. By aggregating the responses in this manner, we aimed to distinguish between negative, neutral, and positive attitudes effectively. The third part covered information on measures taken on the cows during the periparturient period (e.g., surveillance of body temperature). The fourth part documented the presence of pasture access or an outdoor exercise area for the cows (Supplementary 1).

For the purpose of this study, an additional questionnaire was handed to the farm manager to ensure that the information was provided by a person responsible for stockmanship. The respondents’ consent was sought at the introduction of the questionnaire to ensure voluntary and ethical participation. The questionnaire was to be completed in private. It consisted of a modified version of the previously evaluated 24-item Brief HEXACO Inventory (BHI) (45) and was chosen to serve as the foundation to record farmer personality characteristics. As outlined previously (24), the honesty-humility domain was omitted from the assessments because certain statements were deemed potentially compromising for farmers. An overview of the characteristics of each HEXACO-domain is provided in Supplementary 2. Each remaining HEXACO-facet is covered by one statement. Answer choices for every statement are recorded according to the HEXACO Personal inventory Revised (46, 47) on a five-point Likert Scale (44), with its corresponding answer options: Strongly Disagree, Disagree, Neutral, Agree, Strongly Agree (Supplementary 3). Participation was on a voluntary basis and information if a farmer completed this questionnaire was not shared with the study team. Completed as well as not completed forms were handed back to the study team in a closed envelope. Farms were automatically assigned a continuous, pseudonymized ID number within the database. Envelopes from all farms were collected and at different time points, they were opened and transferred to the database by an individual not involved in on-farm data collection.

### 2.3 Bulk tank milk *Fasciola hepatica* and *Ostertagia ostertagi* antibody status

On each farm, the farm manager collected a bulk tank milk (BTM) sample from the central bulk tank as described previously (39, 41, 48). Samples were drawn toward the end of the grazing season (August – October) in order to improve comparability across farms. The presence of *F. hepatica* and *O. ostertagi* was assessed on farm level by determining antibodies in the BTM samples. The IDEXX Fasciolosis Verification Test (IDEXX GmbH) and the Svanovir *O. ostertagi*-Ab ELISA (Boehringer Ingelheim) were used for the detection of antibodies against *F. hepatica* and *O. ostertagi*, respectively (48). In accordance with the manufacturer’s instructions, a threshold of a sample/positive (S/P) ratio > 30% indicated seropositivity for *F. hepatica* and ELISA results for *O. ostertagi* were considered positive at a cut-off value of ≥0.5 Optical Density ratio at 405 nm. This value provides indication that herds probably experience negative impact on milk yield (8).

### 2.4 Data editing and preparation

After each farm visit, data were manually transferred to a central SQL-data base which allowed for plausibility checks of the data. Plausibility of the data was assessed again after exporting datasets from the database. In case of potentially implausible values, data were re-assessed examining the original paper-based sheets as well as the information within the database. Transcription and editing of HEXACO followed the procedures outlined in prior research, adhering to recommendations specific to this field and covering HEXACO regulations (24, 49, 50). For example, if a statement was answered with answer option “strongly disagree,” this corresponds to number 1, “disagree” corresponds to 2, if answer option “neutral” was chosen, it corresponds to number 3, if “agree” was selected, this corresponds to 4, and if “strongly agree” was chosen, it corresponds to number 5. As every surveyed HEXACO-domain (Emotionality, Extraversion, Agreeableness, Conscientiousness, Openness) contains four scores for their corresponding facets, it is necessary to summarize these four scores and divide by four. These calculated means can potentially range from 1 to 5, hence the lowest and highest score an individual can receive for each component is 1 and 5, respectively. Farm-level seropositivity/negativity for *F. hepatica* and *O. ostertagi*, respectively, was expressed as a binary variable based on the aforementioned cut-off values.

### 2.5 Statistical analyses

All statistical analyses were performed in R software for statistical computing version 4.3.1 (51). Elastic net regression was implemented to predict farm-level parasite status based on the available set of covariates available in Supplementary 1, 2, 4, 5 and Tables 1, 2 (52). Elastic net regression represents a powerful classification technique in prediction modeling as it combines the penalties of LASSO (least absolute shrinkage and selection operator), and ridge regression methods and hence helps to overcome their individual limitations: LASSO is able to perform selection of features by shrinking coefficients of less important covariates to zero, removing them from the model. Yet, LASSO is very dependent on the data when selecting features and may hence produce unstable results. Ridge regression on the other hand cannot effect feature selection, but is able to effectively handle multicollinearity and overfitting (52, 53).

**Table 1:** Results of HEXACO questionnaire of 193 German dairy farmers.

HEXACO factor	Mean ± standard deviation	Minimum–Maximum
Emotionality	2.8 ± 0.6	1.3–4.5
Extraversion	3.7 ± 0.5	2.3–4.8
Agreeableness	3.0 ± 0.5	1.5–4.5
Conscientiousness	3.7 ± 0.6	2.0–5.0
Openness	3.4 ± 0.6	1.8–5.0

**Table 2:** Results of the elastic net regression model for *Fasciola hepatica* and *Ostertagia ostertagi*.

Covariate (category)	Model for *F. hepatica*	Model for *O. ostertagi*
	Point estimate^*^	Point estimate^*^
Pasture access	**2.1**	**1.1**
*O. ostertagi* seropositivity	**1.5**	_
*F. hepatica* seropositivity	_	**1.3**
Conscientiousness	**0.2**	−0.05
Farming type (organic farming)	**0.2**	**0.6**
Agreeableness	−0.1	−0.02
Openness	·	·
Extraversion	·	**−0.3**
Emotionality	·	**−0.3**
Emotional relationship (neutral)		·
Emotional relationship (agree)	·	·
Income (supplementary income)	·	·
Satisfaction animal health (neutral)	·	**0.5**
Satisfaction animal health (agree)	·	·
Lameness	·	−0.01
Facial expression (at every calving)	·	·
Facial expression (in conspicuous calving)	·	·
Herd size	·	·
Year 1	·	·
Year 2	·	·
Year 3	·	·
Pressure (neutral)	·	·
Pressure (agree)	·	·
Continuing education (neutral)	·	·
Continuing education (agree)	·	·
Animal handling (neutral)	·	·
Animal handling (agree)	·	·
Care of male calves (agree)	·	·
Patience (neutral)	·	·
Patience (agree)	·	·
Discussions improvement (neutral)	·	0.06
Discussions improvement (agree)	·	−0.1
Pain (neutral)	·	·
Pain (agree)	·	·
Observation behavior (at every calving)	·	·
Observation behavior (at conspicuous calving)	·	·

^*^Larger values of coefficients reflect a stronger influence of the corresponding covariate on the target. Covariates set to zero with no influence on seropositivity are marked by “·”. Values > 0.1 are in bold. Detailed parameter definition is provided in Supplementary 1, 3.

Separate models were built for *F. hepatica* and *O. ostertagi*. As an initial step, the data were split into training data and test data at a ratio of 0.8 to 0.2, respectively. Custom control parameters were determined via 10-fold cross validation, repeated five times. The model was trained in order to obtain the optimum alpha (balance between variable selection and shrinkage) and lambda (overall strength of regularization) values, i.e., the suitable hyperparametrization to create an optimal elastic net model that balances prediction accuracy and interpretability (52, 53). The selection of the best model was based on the highest accuracy value. The final values for the *F. hepatica* model were alpha = 0.7 and lambda = 0.1. With alpha = 0.7, a considerable set of covariates was expected to be set to zero. As for the *O. ostertagi* model, the final values were alpha = 0.3, and lambda = 0.1. Predictions were made on the test data set. Model performance was evaluated by creating a confusion matrix and assessing precision, predictive accuracy, recall, and F1 score.

## 3 Results

### 3.1 Descriptive results

**Figure 1:**
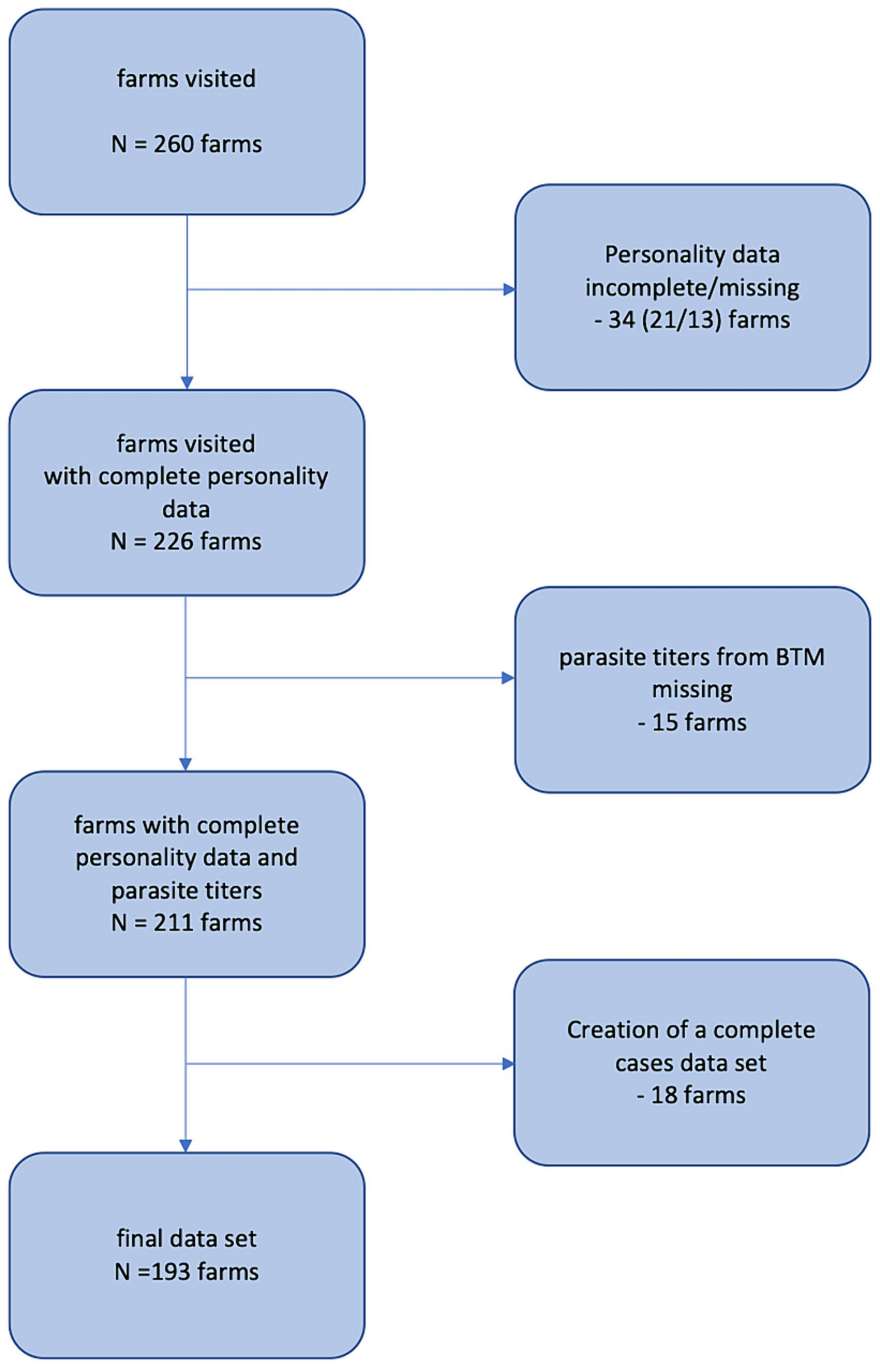
Flowchart for creation of the final dataset, on which elastic net regression analysis was based. A total of 67 farms were excluded for different reasons.

About 6% of all invited farms responded and were visited by the study team. On these 260 inspected dairy farms, 11,539 cows were housed. A complete workflow of how the final dataset for analysis was retrieved is illustrated in Figure 1. Finally, 193 farms with 8,774 cows entered the model building procedure. The mean herd size was 45 (±31) cows, ranging from 5 to 231 animals. The vast majority of farms kept German Simmental (151 farms, 78.2%) breed, followed by mixed-breed (21 farms, 10.9%), Brown Swiss (18 farms, 9.3%) and German Holstein (three farms, 1.6%). On 55 (28.5%) farms, cows were kept in tie stall facilities and on 138 (71.5%) farms in cubicle pens. Access to pasture was available for 68 (35.2%) dairy herds and 33 farms (17.1%) were operated as organic farms. Forty-seven (24.4%) BTM samples were seropositive for *F. hepatica* and 146 (75.6%) were seronegative. For *O. ostertagi*, 77 (39.9%) farms were seropositive and 116 (60.1%) were seronegative. Forty-two (21.8%) samples were seropositive for both endoparasites. Measures against endoparasitic helminths were implemented on 32 farms (16.6%) while 161 farms (83.4%) did not apply anthelmintic treatments.

Results of the HEXACO questionnaire are displayed in Table 1. Scores for conscientiousness (3.71 ± 0.60) and Extraversion (3.71 ± 0.49) are the most pronounced personality traits from farm managers. HEXACO factor Openness (3.38 ± 0.60) also reveals elevated scores. Agreeableness (3.02 ± 0.54) is located almost in the center, while emotionality achieved the lowest values (2.82 ± 0.62) in this evaluation. A comprehensive descriptive statistic of the 193 participating dairy farms can be found in Supplementary 4 for farm characteristics and stockman’s attitude as and in Supplementary 5 for detailed HEXACO data.

### 3.2 Regression analyses

Results of the elastic net regression models are compiled in Table 2. Point estimates of the respective covariates are conveniently interpretable. In the present analyses, estimates <0.1 were not considered sufficiently reliable to infer an influence on the outcome (54).

The *F. hepatica* model performed with a precision of 88.9%, a predictive accuracy of 81.0%, recall of 82.8%, and an F1 score of 85.7%. Four covariates were selected as relevant to predict *F. hepatica* seropositivity. Pasture access was the top-ranking feature (*β* = 2.1) favoring *F. hepatica* seropositivity, followed by *O. ostertagi* seropositivity (*β* = 1.5), higher conscientiousness values (*β* = 0.2), and an organic farming type (*β* = 0.2).

The model performance of the *O. ostertagi* model was as follows: precision 86.4%, predictive accuracy 76.2%, recall 73.1%, F1 score 79.2%. The top-ranking feature was seropositivity for *F. hepatica* (*β* = 1.3), followed by pasture access (*β* = 1.1), organic farming (*β* = 0.6), and neutrality in regard to the farmer’s satisfaction with animal health on their operation (*β* = 0.5). Further relevant features were extraversion (*β* = −0.3) and emotionality (*β* = −0.3).

## 4 Discussion

Given global and widespread occurrence of anthelmintic resistance, effective knowledge transfer and compliance with the implementation as well as adherence to sustainable parasite control programs in livestock are urgently needed in order to ensure animal health and wellbeing. This necessity is emphasized by the fact that the prevalence of helminth infections in cattle remains high despite the available knowledge of parasite epidemiology and potential control measures (20, 34). Evidence has suggested that much of the variability on dairy farms could be explained through more personal traits of the farmers themselves (14, 21, 23). Moreover, individual characteristics and the manner in which a person communicates are connected (45). Hence, it appears plausible that personalized communication strategies are required during veterinary consultation given the differential personality background in order to guarantee client satisfaction, efficient exchange of information ultimately leading to improved adherence to medical guidance (24, 55). According to Vande Velde et al. (14), the adoption of sustainable strategies to combat helminth infection in dairy cows is influenced by a variety of personal factors. Since livestock farming represents a specific lifestyle, a considerable proportion of decision-making can be delineated from personal traits of farmers and elements of their social environment (56–58). The underlying mechanisms necessary to comprehend why recommendations for sustainable intervention strategies are not sufficiently implemented on farm remain largely uncertain and minimal qualitative research has been conducted to acquire a deeper understanding of the mindset, beliefs, motivations, and characteristics of livestock managers (14, 18, 26). Against this background, the aim of the present study was to evaluate to what extent features related to personality traits and attitudes of dairy farmers could be used to predict parasite occurrence expressed as seropositivity of the farms for *F. hepatica* and *O. ostertagi*.

As expected, pasture access was a top-ranking predictor for both *F. hepatica* and *O. ostertagi* positivity. This was an expected outcome, as several aspects of both parasites’ life cycle are related to pasturing grounds. Specific environmental conditions are required by both pathogens to complete their life cycle and to infect definitive hosts which include potential snail habitats for intermediate hosts as well as climatic and topographic conditions ensuring transmission (4, 54, 59). *Ostertagia ostertagi* seropositivity predicted exposure to *F. hepatica* in the *F. hepatica* model and *F. hepatica* seropositivity predicted exposure to *O. ostertagi* in the *O. ostertagi* model. Coinfections with these two parasites are common and widespread in dairy cows (20, 38, 60). As outlined previously, both parasites share central elements in their life cycle that subsequently translate into seropositivity in definitive hosts, i.e., dairy cows due to predisposing on-farm settings. Organic farming also predicted seropositivity for both parasites. This can be explained by aspects often present on organic farms such as grazing of cows and limited use of anthelmintics, which then translates into the transmission of gastrointestinal nematodes and trematodes (61, 62).

In the *F. hepatica* model, a higher conscientiousness score was a predictor for *F. hepatica* seropositivity. Within the HEXACO frameworks, conscientiousness reflects an individual’s tendency toward responsible, goal-directed behavior, organizational skills, and diligence and a higher score refers to a greater presence of these characteristics in a person. Accordingly, conscientiousness is an important dimension influencing behavior, decision-making, and interactions with other people (32, 63, 64). Therefore, it was unexpected that more conscientious farmers may be more prone to *F. hepatica* seropositivity of their cows as higher conscientiousness may lead to better farm management practices that could reduce the risk of parasite exposure in the first place. It is yet important to be aware that very high scores (e.g., ≥4) for conscientiousness may indicate perfectionism or being overly rigid entailing drawbacks in certain regards. More specifically, perfectionism in the context of the perfectionism trap concept, could lead to overemphasis on certain practices while neglecting others, being unable to channel directed interventions, and procrastination or causing stress and burnout (65–67). Highly conscientious farmers may thus focus intensely on specific aspects of farm management while simultaneously missing broader or more strategic procedures like comprehensive parasite control programs. Furthermore, these farmers may be less inclined to delegate tasks believing they must handle everything themselves to ensure correct execution. In the context of farm management, where efficiency and decision-making are critical due to the complexity and plethora of daily tasks, the negative effects of perfectionism on stress management and psychological well-being could impact overall farm management efficiency, especially when dealing with complex issues like bovine fasciolosis with intricate epidemiological dynamics. On the other hand, a higher conscientiousness score may act as a proxy for other aspects that actually favors farm-level seropositivity of dairy cows such as environmental conditions, geographical location, or management regimes. These factors may well be in action despite the farmer’s conscientiousness. Additionally, more conscientious farmers could well provide animals with housing conditions, e.g., including pasture with snail habitats, which actually translate into a higher infection risk for *F. hepatica*. Whereas conscientious farmers may in general be more aware of hygiene, control strategies, or pasture management, the exposure to *F. hepatica* may thus be influenced by multiple complex aspects beyond the control of conscientious behavior *per se*. These potential relationships of conscientiousness with other variables require further investigation to fully understand the underlying mechanisms.

Higher farmer agreeableness was inversely associated with *F. hepatica* seropositivity, suggesting that farms managed by more agreeable farmers may be less likely to seropositive for *F. hepatica*. Agreeableness within the HEXACO model, encompasses characteristics such as cooperativeness, kindness, and a tendency to avoid conflict. Higher scores in agreeableness suggest more cooperative and amicable behavior (28, 32). In practical terms, agreeable farmers may engage more effectively in targeted preventive and management practices, such as regular deworming, better pasture management, and overall improved herd health protocols in the context of an integrated control strategy, reducing the prevalence of *F. hepatica*.

Neutrality in regard to a farmer’s satisfaction with the animal health situation on their operations appeared to be a predictor for *O. ostertagi* seropositivity. The neutrality or lack of strong satisfaction regarding animal health might indicate neglect or insufficient attention as a result of indifference to animal health. Moreover, such farmers may not prioritize preventive control measures which may support inadequate intervention strategies, lack of veterinary care or reluctance to seek professional advice. Secondly, farmers that are not actively satisfied with animal health may be less vigilantly watching their animals for signs of disease or discomfort and pathological conditions may go unnoticed. Furthermore, a lack of clear satisfaction with animal health might reflect overall deficiencies in management, delayed or inconsistent treatment as well as neglect of environmental factors that contribute to the transmission of gastrointestinal nematodes. In conclusion, a farmer’s neutral stance on the animal health situation may indicate a lack of proactivity to tackle issues present on farm or reduced attention to preventive measures for gastrointestinal nematodes. Yet, on the other hand, neutrality in regard to animal health may also simply refer to the fact that a farmer is overall unconcerned or neither exceedingly satisfied nor considerably unhappy about the health situation on their farm.

Higher values for emotionality where inversely associated with predicting *O. ostertagi* seropositivity. Farmers attaining higher scores for emotionality may be more open to intense emotions, empathy, compassion, or creativity. Forming emotional relationships with other beings may be more important to these farmers. On the other hand, they may be more prone to anxiety, worry, or stress (28, 31, 32). Consequently, more emotional farmers may be more empathetic and sensitive to their cows’ needs assigning a higher priority to attentive surveillance of the animals as well as to the early detection of health issues. Moreover, the implementation of preventive strategies and health control interventions may be pursued more diligently by more emotional farmers. The attentiveness and sensibility of farmers scoring higher for emotionality may be reflected by lower prevalence of gastrointestinal nematodes on their farms and an overall lower risk for seropositivity.

Higher extraversion turned out to have an inverse relationship with *O. ostertagi* seropositivity as well. Extraversion refers to an individual’s inclination to engage in social interactions and to enjoy being surrounded by others. A person attaining high values in this domain tends to be outgoing, cheerful, energetic, and thriving in social settings. Moreover, particularly extraverted people usually have a higher activity level and are comfortable communicating their true opinions and assuming a leadership role. Of particular interest is their distinct propensity to embrace novel experiences and to engage in risk-taking behaviors (28, 31, 32). Accordingly, more extraverted farmers may in general be more open to recent scientific developments regarding sustainable parasite control and hence take a proactive approach to addressing health issues. As a consequence, control measures to combat gastrointestinal nematodes may be composed of different complementary strategies that together result in an effective management of the epidemiological situation on the respective farms. Moreover, extraverted farmers may be more drawn to closely observing their animals and interacting with their cows. This increased attention and familiarity with the individual animal may improve early detection of health problems leading to quicker and more effective intervention.

Both the model for *F. hepatica* seropositivity as well as the model for *O. ostertagi* seropositivity selected only two common covariates, i.e., pasture access and organic farming, both biologically intertwined in their relevance. Furthermore, seropositivity of one parasite was a strong predictor for the other parasite in both models. Even though coinfections with both parasites are common and both parasites share central aspects in their epidemiology such as the dependence of transmission on several management-related or weather and climate associated features (4, 38, 54), each of the parasites has unique characteristics in regard to their life cycle, stages infective for the definitive host, and transmission dynamics that need to be taken into account when designing control measures. For example, regarding *Fasciola hepatica*, the intermediate host is a crucial element to be considered in any control strategy, whereas *O. ostertagi* has a direct life cycle (54). Hence control strategies against *F. hepatica* are complex, labor intensive, and time-consuming, requiring considerable commitment. Some farmers may be more inclined and determined to follow such intervention strategies, while others may be more reluctant to dedicate large amounts of resources. Moreover, shared aspects in epidemiology of both parasites may exert an influence on both, whereas others instead more profoundly affect one parasite due to their specific biology. These aspects could thus be the reason for differences in both models and even result in the opposite influence of certain covariates on the outcome depending on the target variable. Moreover, the importance of some features could simply differ among both outcomes with some predictors playing a more central role regarding parasite occurrence and exposure in dairy cows. Dairy cow helminth infections represent a complex setting that is influenced by a variety of factors related to farm characteristics, environment, climate, parasite biology, host species, and other extrinsic and intrinsic elements (38, 54, 68). Depending on the underlying setting, these factors may be in varying interaction which subsequently translates into differential exposure of the host. Understanding these variations is essential for comprehensively studying and forecasting the occurrence of parasitic infections. This may encompass additional data collection or taking into account specific features linked to the biology and ecology of each parasite.

It is crucial to be aware of the limitations of this study in order to correctly interpret the results. German data protection legislation prohibits obtaining the contact information of dairy farms *a priori*. Therefore, the farm recruitment process could not be based on random sampling but had to rely on voluntary participation and hence self-selection of farmers. Due to the voluntary participation strategy to recruit farmers, a certain level of selection bias is likely, since individuals who willingly participated may have had a higher motivation, interest, awareness or proactivity (69). Farmers with animal health problems hence may have been more inclined to get involved. Yet on the other hand, proactive farmers with an overall better animal health situation on their operations compared with the entirety of the target population may be overrepresented. Self-selection bias may be specifically relevant when answering questions on personality traits, attitudes, and beliefs. This may furthermore be complemented by social desirability bias, since farmers may tend to answer questions as they think they are supposed to rather than providing their true beliefs (70, 71). This may be the reason why many of the attitude-related questions as well as the personality aspects were characterized by a low variability within the data and thus potential relevance of these factors may have remained hidden. Given these limitations, the external validity and generalizability of the results could be compromised limiting the ability to extrapolate results to a broader population. Moreover, the present work may not entirely reflect the true beliefs and practices of the farmers or of the underlying target population. This frequently is the inherent limitation of qualitative research since the true beliefs or thoughts of the participants remain inaccessible. Yet taking into account the results of the present work and interpreting the outcomes against the contextual background of the study, it is even more interesting to see the relevance of personality and attitude-related covariates in regard to parasite occurrence. Moreover, assuming that farmers with improved conditions on their farms as well as those with animal health issues may have been more motivated to get enrolled, this could mitigate the selection bias.

We used the 24-item BHI (45) to record personality traits of the farmers. In the context of the present work, it was not possible to distribute the full-length inventory since the farm visits were fairly elaborate and required the farmers’ attention and engagement. Furthermore, the full-length HEXACO inventory may have decreased acceptability. According to the authors (24, 45), the BHI had been specifically designed for exploratory research in large scale studies and has been shown to establish the validity of the original HEXACO personality inventory revised with a fairly high level of accuracy (24, 45). Moreover, the BHI was the preferred personality measurement framework, because it covers the breadth and the high and low poles of each domain appropriately (45) and it follows principles for short measures of personality (45, 72) better than Ten Item Personality Measure (73). In the context of the current work, we hence are confident to have captured the essence of farmer personality characteristics. This assumption is particularly emphasized by comparison of HEXACO results with a previously conducted study by Schröter and Mergenthaler (49). Their online survey was conducted among 240 German livestock farmers and yielded high scores for the personality traits conscientiousness (3.68 ± 0.62) and extraversion (3.72 ± 0.57) as in our trail. Same to our study, emotionality produced the lowest values (2.74 ± 0.61).

Farmers are confronted with complex risk assessments in order to make decisions for the control of health problems on their farms. Their choices are not solely based on economic considerations and feasibility but to a considerable extent dependent on intrinsic, socio-psychological factors and individual characteristics (27). The present work has shed light on the relevance of personality-associated traits of farmers that could be relevant for understanding complex disease situations such as parasitic infections. Based on the acquired understanding of farmer traits, communication styles of veterinary consultants can be adapted to match the client and to promote self-motivation and encouragement of stakeholders. In a next step, more detailed investigations are necessary to understand the underlying mechanisms better and to develop tailored strategies for communication, intervention, and general consultation.

## Data Availability

The datasets presented in this article are not readily available because personality and attitude data were individually collected from dairy farmers, each providing written consent with the understanding that data would not be transferred to third parties. Therefore, data transfer requires an additional formal contract. Qualified researchers can access data by signing a contract with the University of Veterinary Medicine Hannover, ensuring data confidentiality per German law. Currently, no data access committee exists, but one will be established, comprising authors, University of Veterinary Medicine Hannover members, and funding institution representatives. Cooperative partners meeting contract requirements may inquire at: lothar.kreienbrock@tiho-hannover.de. Requests to access the datasets should be directed to lothar.kreienbrock@tiho-hannover.de.
