# Supplementary material for: Animal health as a function of farmer personality and attitude: using the HEXACO model of personality structure to predict farm-level seropositivity for Fasciola hepatica and Ostertagia ostertagi in dairy cows
Source: Front Vet Sci. 2024 Oct 2;11:1434612. doi: 10.3389/fvets.2024.1434612 (PMC11479864; doi:10.3389/fvets.2024.1434612)
Supplement: Supplementary file 1 [file Data_Sheet_1.pdf]

Supplementary Material

Supplementary 1 Surveilled factors and answer options

	Variable	Variable labelling	Answer options
Farm characteristics	Housing system[a]		Cubicle pen<br>Tie stall<br>other
	Pasture	Pasture access present	Access to pasture<br>No Access to pasture
	Farming type	Farming type	Conventional farming<br>organic farming
	Herd size	Herd size	Number of cows
	Lameness	Lameness	Prevalence of lame cows
	Income Type	Income	Dairy farming as main income<br>Dairy farming as supplementary income
	Visiting year	Year	Year of farm visit
Stockman's attitude	I am satisfied with the health of my animals.	Satisfaction animal health	1 Disagree<br>2 Neutral<br>3 Agree
	I feel overwhelmed by the daily work of dairy cow husbandry.	Pressure	1 Strongly Disagree<br>2 Disagree<br>3 Neutral

		4 Agree<br>5 Strongly Agree
I can imagine establishing an emotional relationship with a cow.	Emotional relationship	1 Strongly Disagree<br>2 Disagree<br>3 Neutral<br>4 Agree<br>5 Strongly Agree
I regularly attend professional events of continuing education.	Continuing education	1 Strongly Disagree<br>2 Disagree<br>3 Neutral<br>4 Agree<br>5 Strongly Agree
Dealing with cows is easy for me.	Animal handling	1 Strongly Disagree<br>2 Disagree<br>3 Neutral<br>4 Agree<br>5 Strongly Agree
I accept economic losses in order to ensure adequate care for bull calves.	Care of Male calves	1 Strongly Disagree<br>2 Disagree<br>3 Neutral<br>4 Agree<br>5 Strongly Agree
It is important for me to be patient with my cows.	Patience	1 Strongly Disagree<br>2 Disagree<br>3 Neutral

			4 Agree<br>5 Strongly Agree
	It affects me to see a cow in pain.	Pain	1 Strongly Disagree<br>2 Disagree<br>3 Neutral<br>4 Agree<br>5 Strongly Agree
	On our farm, we often discuss what we could improve about the way we keep dairy cows	Discussions improvement	1 Strongly Disagree<br>2 Disagree<br>3 Neutral<br>4 Agree<br>5 Strongly Agree
Calving practices	I look at the cow's facial expression and eyes.	Facial expression	1 At every calving<br>2 At conspicuous calving<br>3 Never/rarely
	I observe the general behavior of the cow.	Observation behaviour	1 At every calving<br>2 At conspicuous calving<br>3 Never/rarely

[a] for descriptive statistics only and excluded from modelling process

Supplementary 2 HEXACO-domains with Facets and Common Defining Adjectives

HEXACO-domain	Facet	Common Defining Adjectives	
Emotionality	Fearfulness<br>Anxiety<br>Dependence<br>Sentimentality	emotional, oversensitive, sentimental, fearful, anxious, vulnerable	brave, tough, independent, self-assured, stable
Extraversion	Social self-esteem<br>Social boldness<br>Sociability<br>Liveliness	outgoing, lively, extraverted, sociable, talkative, cheerful, active	shy, passive, withdrawn, introverted, quiet, reserved
Agreeableness	Forgivingness<br>Gentleness<br>Flexibility<br>Patience	patient, tolerant, peaceful, mild, agreeable, lenient, gentle	ill-tempered, quarrelsome, stubborn, choleric
conscientiousness	Organization<br>Diligence<br>Perfectionism<br>Prudence	organized, disciplined, diligent, careful, thorough, precise	sloppy, negligent, reckless, lazy, irresponsible, absent-minded
Openness to experience	Aesthetic appreciation<br>Inquisitiveness<br>Creativity<br>Unconventionality	intellectual, creative, unconventional, innovative, ironic	shallow, unimaginative, conventional

Supplementary 3 HEXACO questionnaire. Answer options are “Strongly agree”, “agree”, “neutral”, “disagree”, “strongly disagree” for all statements.

HEXACO-factor	Statement according to (44)	Facet
Openness to experience	I can look at a painting for a long time.	Aesthetic Appreciation
Conscientiousness	I make sure things are in the right spot.	Organization
Agreeableness	I remain unfriendly to someone who was mean to me.	Forgivingness
Extraversion	Nobody likes talking with me.	Social Self-Esteem
Emotionality	I am afraid of feeling pain.	Fearfulness
Openness to experience	I think science is boring.	Inquisitiveness
Conscientiousness	I postpone difficult tasks as long as possible.	Diligence
Agreeableness	I often express criticism.	Gentleness
Extraversion	I easily approach strangers.	Sociability
Emotionality	I worry less than others.	Anxiety
Openness to experience	I have a lot of imagination.	Creativity
Conscientiousness	I work very precisely.	Perfectionism
Agreeableness	I tend to quickly agree with others.	Flexibility
Extraversion	I like to talk with others.	Social Boldness
Emotionality	I can easily overcome difficulties on my own.	Dependence
Openness to experience	I like people with strange ideas.	Unconventionality
Conscientiousness	I often do things without really thinking.	Prudence
Agreeableness	Even when I’m treated badly, I remain calm.	Patience
Extraversion	I am seldom cheerful.	Liveliness
Emotionality	I have to cry during sad or romantic movies.	Sentimentality

Supplementary 4 Farm characteristics and stockman's attitude

	Predictor	Categories	n_{farms} (%)	n_{cows} (%)
Farm characteristics	Housing system	Tie stall	55 (28.5)	1,306 (14.9)
		Cubicle pen	138 (71.5)	7468 (85.1)
	Pasture access present	Access	68 (35.2)	2283(26.0)
		No access	125 (64.8)	6,491 (74.0)
	Farming type	Conventional	160 (82.9)	7,699 (87.7)
		Organic	33 (17.1)	1,075 (12.3)
	Herd size	numeric value	45 ± 31; 5 (min) – 231 (max)	
	Lameness	numeric value	25% ± 14.0%; 3.3% (min) – 86.6% (max)	
	Income	Main income	157 (81.3)	8,029 (91.5)
		Supplementary income	36 (18.7)	745 (8.5)
	Year	2016	1 (0.5)	25 (0.3)
		2017	78 (40.4)	4,322 (49.3)
		2018	74 (38.3)	3,182 (36.2)
		2019	40 (20.7)	1,245 (14.2)
BTM – antibody status	*F. hepatica*	Seropositive	47 (24.4)	7,294 (83.1)
		Seronegative	146 (75.6)	1,480 (16.9)
	O. ostertagi	Seropositive	77 (39.9)	3,065 (34.9)
		Seronegative	116 (60.1)	5,709 (65.1)
Stockman's attitude	Satisfaction animal health	1	24 (12.4)	1,241 (14.1)
		2	36 (18.7)	1,527 (17.4)
		3	133 (68.9)	6,006 (68.5)

	Pressure	1	96 (49.7)	4,032 (46.0)
		2	48 (24.9)	2,236 (25.5)
		3	49 (25.4)	2,506 (28.5)
	Emotional relationship	1	23 (11.9)	1,354 (15.4)
		2	20 (10.4)	1,015 (11.6)
		3	150 (77.7)	6,405 (73.0)
	Continuing education	1	17 (8.8)	610 (6.9)
		2	24 (12.4)	850 (9.7)
		3	152 (78.8)	7,314 (83.4)
	Animal handling	1	7 (3.6)	283 (3.2)
		2	13 (6.7)	664 (7.6)
		3	173 (89.7)	7,827 (89.2)
	Care of male calves	1	2 (1.0)	296 (3.4)
		2	0 (0.0)	0 (0.0)
		3	191 (99.0)	8,478 (96.6)
	Patience	1	2 (1.0)	41 (0.5)
		2	11 (5.7)	870 (9.9)
		3	180 (93.3)	7,863 (89.6)
	Pain	1	2 (1.0)	44 (0.5)
		2	3 (1.6)	115 (1.3)
		3	188 (97.4)	8,615 (98.2)
	Discussions	1	10 (5.2)	292 (3.3)
	improvement	2	13 (6.7)	453 (5.2)
		3	170 (88.1)	8,029 (91.5)
Calving practice	Facial expression	1	75 (38.9)	3,627 (41.3)

	2	40 (20.7)	1,753 (20.0)
	3	78 (40.4)	3,394 (38.7)
Observation behavior	1	183 (94.8)	7,976 (90.9)
	2	9 (4.7)	737 (8.4)
	3	1 (0.5)	61 (0.7)

Supplementary 5 Results of the HEXACO questionnaire

HEXACO-factor	Item according to (44)	Answer option	n_{farms} (%)	n_{cows} (%)
Emotionality	I am afraid of feeling pain.	1	27 (14.0)	1,105 (12.7)
		2	51 (26.4)	2,630 (29.9)
		3	41 (21.2)	1,834 (20.9)
		4	59 (30.6)	2,651 (30.1)
		5	15 (7.8)	554 (6.4)
	I worry less than others.	1	15 (7.8)	880 (10.0)
		2	49 (25.4)	1,959 (22.4)
		3	59 (30.6)	2,562 (29.1)
		4	54 (28.0)	2,663 (30.4)
		5	16 (8.3)	710 (8.1)
	I can easily overcome	1	19 (9.8)	1,067 (12.2)
	difficulties on my own.	2	92 (47.7)	4,012 (45.6)
		3	63 (32.6)	2,892 (32.9)
		4	17 (.8)	762 (8.8)
		5	2 (1.0)	41 (0.5)
	I have to cry during sad or	1	33 (17.1)	1,586 (18.1)
	romantic movies.	2	39 (20.2)	1.738 (19.90)
		3	55 (28.5)	2,440 (27.7)
		4	48 (24.9)	2,304 (26.2)
		5	18 (9.3)	706 (8.1)
Extraversion	Nobody likes talking with	1	0 (0.0)	0 (0.0)
	me.	2	1 (0.5)	95 (1.1)
		3	23 (11.9)	918 (10.5)

		4	87 (45.1)	4,158 (47.3)
		5	82 (42.5)	3603 (41.1)
	I easily approach strangers.	1	3 (1.6)	266 (3.0)
		2	31 (16.1)	1,525 (17.4)
		3	54 (28.0)	2,378 (27.1)
		4	77(39.9)	3,401 (38.7)
		5	28 (14.5)	1,204 (13.8)
	I like to talk with others.	1	1 (0.5)	30 (0.4)
		2	6 (3.1)	1192 (2.2)
		3	33 (17.1)	1,689 (19.3)
		4	113 (58.5)	5,295 (60.3)
		5	40 (20.7)	1,568 (17.8)
	I am seldom cheerful.	1	8 (4.1)	472 (5.5)
		2	50 (25.9)	2,299 (26.1)
		3	61 (31.6)	2,371 (27.1)
		4	63 (32.7)	3,014 (34.3)
		5	11 (5.7)	618 (7.0)
Agreeableness	I remain unfriendly to someone who was mean to me.	1	12 (6.2)	675 (7.8)
		2	47 (24.4)	2,139 (24.3)
		3	81 (42.0)	3,648 (41.5)
		4	43 (22.2)	1,974 (22.5)
		5	10 (5.2)	338 (3.9)
	I often express criticism.	1	11 (5.7)	534 (6.2)
		2	53 (27.5)	2,372 (27.0)
		3	79 (40.9)	3,361 (38.3)

		4	44 (22.8)	2,164 (24.6)
		5	6 (3.1)	343 (3.9)
	I tend to quickly agree with others.	1	2 (1.0)	128 (1.5)
		2	35 (18.2)	1,511 (17.2)
		3	100 (51.8)	4,673(53.1)
		4	52 (26.9)	2,316 (26.3)
		5	4 (2.1)	146 (1.7)
	Even when I’m treated badly, I remain calm.	1	11 (5.7)	639 (7.3)
		2	46 (23.8)	2,051 (23.3)
		3	55 (28.5)	2,546 (28.9)
		4	69(35.8)	2,950 (33.5)
		5	12 (6.2)	588 (6.7)
Conscientiousness	I make sure things are in the right spot.	1	0 (0.0)	0 (0.0)
		2	15 (7.8)	905 (10.3)
		3	40 (20.7)	1,775 (20.3)
		4	85 (44.0)	3,787 (43.2)
		5	53 (27.5)	2,307 (26.2)
	I postpone difficult tasks as long as possible.	1	6 (3.1)	203 (2.3)
		2	38 (19.7)	1,541 (17.6)
		3	53 27.5)	2,588 (29.5)
		4	77 (39.9)	3,725 (42.3)
		5	19 (9.8)	717 (8.3)
	I work very precisely.	1	1 (0.5)	102 (1.2)
		2	9 (4.7)	584 (6.7)
		3	47 (24.4)	2,097 (23.9)

		4	96 (49.7)	4,435 (50.5)
		5	40 (20.7)	1,556 (17.7)
	I often do things without really thinking.	1	3 (1.6)	148 (1.7)
		2	15 (7.8)	647 (7.5)
		3	44 (22.8)	1,916 (21.8)
		4	97 (50.3)	4397 (50.1)
		5	34 (17.6)	1,666 (18.9)
Openness	I can look at a painting for a long time.	1	12 (6.2)	615 (7.0)
		2	53 (27.5)	2,732 (31.1)
		3	79 (40.9)	3,272 (37.3)
		4	43 (22.3)	1,967 (22.4)
		5	6 (3.1)	188 (2.1)
	I think science is boring.	1	5 (2.6)	161 (1.8)
		2	10 (5.2)	428 (4.9)
		3	38 (19.6)	1,769 (20.2)
		4	81 (42.0)	3,695 (42.1)
		5	59 (30.6)	2,721 (31.0)
	I have a lot of imagination.	1	1 (0.5)	199 (2.3)
		2	20 (10.4)	893 (10.2)
		3	73 (37.8)	3,191 (36.4)
		4	69 (35.8)	3034 (34.5)
		5	30 (15.5)	1,457 (16.6)
	I like people with strange ideas.	1	9 (4.7)	508 (5.8)
		2	33 (17.1)	1,603 (18.2)
		3	81 (42.0)	3,243 (36.9)

	4	57 (29.5)	2,914 (33.1)
	5	13 (6.7)	506 (5.8)
