# Supplementary material for: Animal health as a function of farmer personality and attitude: using the HEXACO model of personality structure to predict farm-level seropositivity for Fasciola hepatica and Ostertagia ostertagi in dairy cows
Source: Front Vet Sci. 2024 Oct 2;11:1434612. doi: 10.3389/fvets.2024.1434612 (PMC11479864; doi:10.3389/fvets.2024.1434612)
Supplement: Supplementary file 2 [file Table_1.docx]

Supplementary Material

Table 1. Results of HEXACO questionnaire of 193 German dairy farmers

| HEXACO Factor | Mean ± standard deviation | Minimum – Maximum |
| --- | --- | --- |
| Emotionality | 2.8 ± 0.6 | 1.3 – 4.5 |
| Extraversion | 3.7 ± 0.5 | 2.3 – 4.8 |
| Agreeableness | 3.0 ± 0.5 | 1.5 – 4.5 |
| Conscientiousness | 3.7 ± 0.6 | 2.0 – 5.0 |
| Openness | 3.4 ± 0.6 | 1.8 – 5.0 |

Table 2. Results of the elastic net regression model for *Fasciola hepatica* and *Ostertagia ostertagi*

| Covariate | Model for *F.hepatica* | Model for *O. ostertagi* |
| --- | --- | --- |
|  | Point estimate^*^ | Point estimate^*^ |
| Pasture access | **2.1** | **1.1** |
| *O. ostertagi* seropositivity | **1.5** | _ |
| *F. hepatica* seropositivity | _ | **1.3** |
| Conscientiousness | **0.2** | -0.05 |
| Farming type (organic farming) | **0.2** | **0.6** |
| Agreeableness | **-0.1** | -0.02 |
| Openness | . | . |
| Extraversion | . | **-0.3** |
| Emotionality | . | **-0.3** |
| Emotional relationship (neutral) |  | . |
| Emotional relationship (agree) | . | . |
| Income (supplementary income) | . | . |
| Satisfaction animal health (neutral) | . | **0.5** |
| Satisfaction animal health (agree) | . | . |
| Lameness | . | -0.01 |
| Facial expression (at every calving) | . | . |
| Facial expression (in conspicuous calving) | . | . |
| Herd size | . | . |
| Year 1 | . | . |
| Year 2 | . | . |
| Year 3 | . | . |
| Pressure (neutral) | . | . |
| Pressure (agree) | . | . |
| Continuing education (neutral) | . | . |
| Continuing education (agree) | . | . |
| Animal handling (neutral) | . | . |
| Animal handling (agree) | . | . |
| Care of male calves (agree) | . | . |
| Patience (neutral) | . | . |
| Patience (agree) | . | . |
| Discussions improvement (neutral) | . | 0.06 |
| Discussions improvement (agree) | . | -0.1 |
| Pain (neutral) | . | . |
| Pain (agree) | . | . |
| Observation behavior (at every calving) | . | . |
| Observation behavior (at conspicuous calving) | . | . |

^*^ Larger values of coefficients reflect a stronger influence of the corresponding covariate on the target. Covariates set to zero with no influence on seropositivity are marked by “.”. Values > 0.1 are in bold.

**
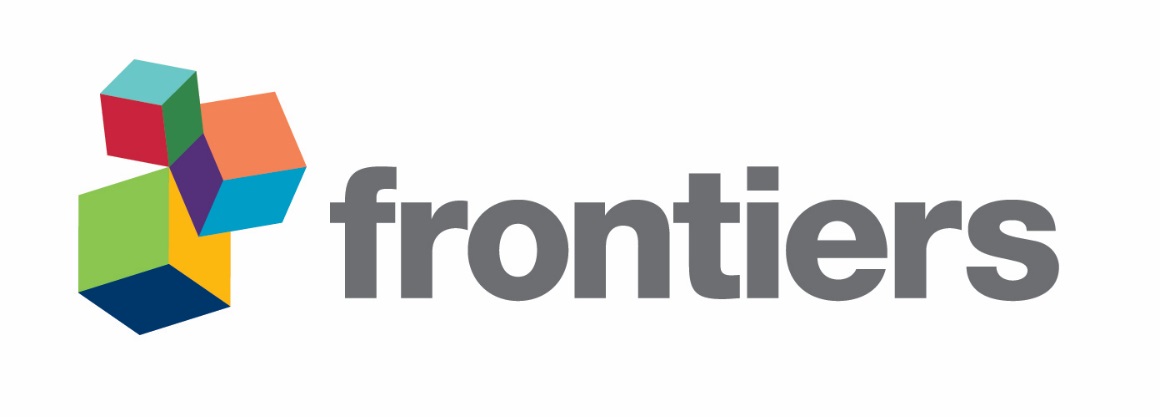
**
